# Imaging evolution of the primate brain: the next frontier?

**DOI:** 10.1016/j.neuroimage.2020.117685

**Published:** 2021-01-05

**Authors:** Patrick Friedrich, Stephanie J. Forkel, Céline Amiez, Joshua H. Balsters, Olivier Coulon, Lingzhong Fan, Alexandros Goulas, Fadila Hadj-Bouziane, Erin E. Hecht, Katja Heuer, Tianzi Jiang, Robert D. Latzman, Xiaojin Liu, Kep Kee Loh, Kaustubh R. Patil, Alizée Lopez-Persem, Emmanuel Procyk, Jerome Sallet, Roberto Toro, Sam Vickery, Susanne Weis, Charles R. E. Wilson, Ting Xu, Valerio Zerbi, Simon B. Eickoff, Daniel S. Margulies, Rogier B. Mars, Michel Thiebaut de Schotten

**Affiliations:** a Brain Connectivity and Behaviour Laboratory, Sorbonne Universities, Paris, France; b Groupe d’Imagerie Neurofonctionnelle, Institut des Maladies Neurodégénératives-UMR 5293, CNRS, CEA, University of Bordeaux, Bordeaux, France; c Centre for Neuroimaging Sciences, Department of Neuroimaging, Institute of Psychiatry, Psychology and Neuroscience, King’s College London, London, United Kingdom; d Institute of Systems Neuroscience, Medical Faculty, Heinrich-Heine University Düsseldorf, Germany; e Institute of Neuroscience and Medicine (Brain & Behaviour, INM-7), Research Center Jülich, Germany; f Frontlab, Institut du Cerveau et de la Moelle épinière (ICM), UPMC UMRS 1127, Inserm U 1127, CNRS UMR 7225, Paris, France; g Wellcome Centre for Integrative Neuroimaging, Centre for Functional MRI of the Brain (FMRIB), Nuffield Department of Clinical Neurosciences, John Radcliffe Hospital, University of Oxford, Oxford, United Kingdom; h Donders Institute for Brain, Cognition and Behaviour, Radboud University Nijmegen, Nijmegen, Netherlands; i Department of Psychology, Royal Holloway University of London, United Kingdom; j Department of Psychology, Georgia State University, Atlanta, United States; k Child Mind Institute, New York, United States; l Neural Control of Movement Lab, Department of Health Sciences and Technology, ETH Zurich, Zurich, Switzerland; m Institute of Computational Neuroscience, University Medical Center Hamburg-Eppendorf, Hamburg University, Hamburg, Germany; n Department of Human Evolutionary Biology, Harvard University, Cambridge, MA, United States; o Univ Lyon, Université Lyon 1, Inserm, Stem Cell and Brain Research Institute, U1208 Bron, France; p Institut de Neurosciences de la Timone, Aix Marseille Univ, CNRS, UMR 7289, Marseille, France; q Institute for Language, Communication, and the Brain, Aix-Marseille University, Marseille, France; r Wellcome Centre for Integrative Neuroimaging, Department of Experimental Psychology, University of Oxford, Oxford, United Kingdom; s Brainnetome Center and National Laboratory of Pattern Recognition, Institute of Automation, Chinese Academy of Sciences, Beijing 100190, China; t CAS Center for Excellence in Brain Science and Intelligence Technology, Institute of Automation, Chinese Academy of Sciences, Beijing 100190, China; u The Queensland Brain Institute, University of Queensland, Brisbane QLD 4072, Australia; v Lyon Neuroscience Research Center, ImpAct Team, INSERM U1028, CNRS UMR5292, Université Lyon 1, Bron, France; w Center for Research and Interdisciplinarity (CRI), Université de Paris, Inserm, Paris 75004, France; x Max Planck Institute for Human Cognitive and Brain Sciences, Leipzig, Germany; y Neuroscience department, Institut Pasteur, UMR 3571, CNRS, Université de Paris, Paris 75015, France; z Integrative Neuroscience and Cognition Center (UMR 8002), Centre National de la Recherche Scientifique (CNRS) and Université de Paris, 75006, Paris, France

## Abstract

Evolution, as we currently understand it, strikes a delicate balance between animals’ ancestral history and adaptations to their current niche. Similarities between species are generally considered inherited from a common ancestor whereas observed differences are considered as more recent evolution. Hence comparing species can provide insights into the evolutionary history. Comparative neuroimaging has recently emerged as a novel subdiscipline, which uses magnetic resonance imaging (MRI) to identify similarities and differences in brain structure and function across species. Whereas invasive histological and molecular techniques are superior in spatial resolution, they are laborious, post-mortem, and oftentimes limited to specific species. Neuroimaging, by comparison, has the advantages of being applicable across species and allows for fast, whole-brain, repeatable, and multi-modal measurements of the structure and function in living brains and post-mortem tissue. In this review, we summarise the current state of the art in comparative anatomy and function of the brain and gather together the main scientific questions to be explored in the future of the fascinating new field of brain evolution derived from comparative neuroimaging.

## Introduction

**Fig. 1:**
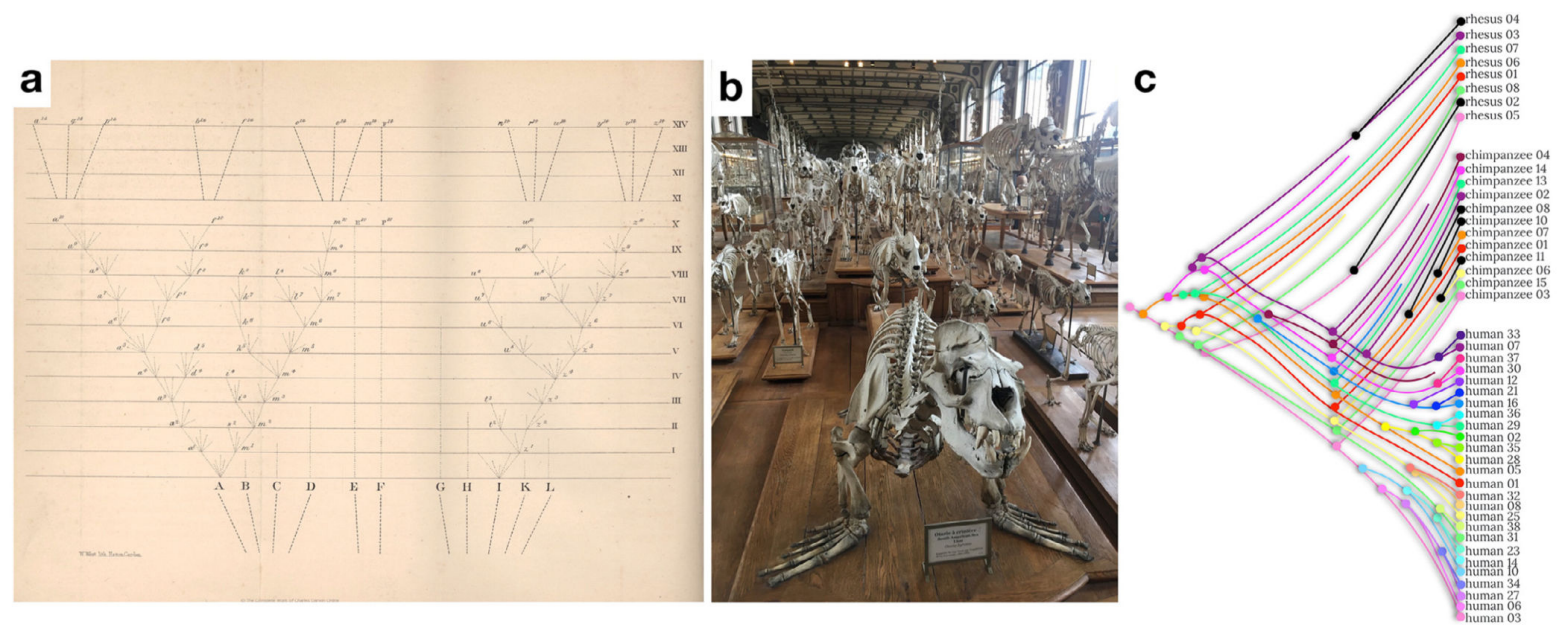
Comparative anatomy as a glimpse at the evolution of species. a) First evolutionary tree (courtesy of © The Complete Work of Charles Darwin Online) as depicted in the 6th edition of the origin of species (Darwin 1859), b) Comparative anatomy of the skeletal structure whereby obvious similarities can be found between a sea lion and a cheetah, suggesting a close common ancestor (Rybczynski et al., 2009, picture taken at the Museum National D’Histoire Naturelle in Paris) c) Example of comparative genetics (limited to the Preferentially Expressed Antigen In Melanoma – PRAME – gene cluster) whereby the evolutionary tree combine within (i.e. interindividual variability in genetics) and between species (comparative genetics) differences (modified from Gibbs et al., 2007). Hue level differences have been coded so that it represents the level of difference with the original phylogenetic branch (in pink). New non-human variations have been coloured in black.

Our brain is the fruit of billions of years of evolution. Evolution, as we currently understand it, strikes a delicate balance between animals’ ancestral history and adaptations to their current niche. Within each generation, discreet changes can occur across phenotypes mostly through genetic recombination (Hirsch 1963). If disadvantageous, these changes are more likely eliminated by natural selection (i.e. survival and reproduction). Accordingly, it is generally assumed that similarities between species are inherited from a common ancestor whereas observed differences are more recent occurrences (Darwin 1859; Fig. 1a). Hence comparing species gives us insight into evolutionary history, and has been applied in multiple fields where precise quantitative measurements are easy to access in large numbers including, for example skeletal structure (e.g. Dutel et al., 2019, see also Fig. 1b) or genetics (e.g. Boffelli et al., 2003; Clifen et al., 2003, see also Fig. 1c)

Thus far, however, large numbers of observations and precise quantitative measurements are lacking for the brain across species due to its fragile, ephemeral and complex organisation. While we know a great deal about the evolution of species, the aforementioned difficulty to work with brain data has hampered progress in our understanding of the evolution of the brain. Better understanding evolution will allow for targeted studies with animal models matching the brain mechanisms in the human to its phylogenetic counterpart. Further, it may also help discover neuroprotective mechanisms allowing for resilience to disease in animals. Recent advancements in neuroimaging, with regard to both hardware and software as well as larger cohort datasets are now opening the door to embark on this new adventure of comparative brain evolution.

Brains differ in many respects across species (Haug, 1987; Stephan, 1975; Ariëns Kappers, 1909) and MRI can compare most of these levels digitally at moderate costs (Krubitzer and Kaas, 2005; Mars et al., 2018). With the advent of better hardware and higher-resolution magnetic resonance imaging sequences that allow researchers to characterise many different aspects of the same brain’s structure and function, it has become feasible to compare different species using a non-invasive repeatable multimodal method of investigation (Thiebaut de Schotten, Croxson and Mars, 2019). Another striking advantage of using magnetic resonance imaging for comparative studies is its feasibility to study large cohorts longitudinally as there is no need to sacrifice animals. Thereby, brains can be manipulated, and the effects of aging, training or lesions can be compared not only within but also between species. Finally, the non-invasive nature of the methods facilitates functional studies that better elucidate brain-behaviour interactions. Amongst the most commonly used MRI sequences to probe the structure of the brain are T1-weighted and T2-weighted scans to visualise different brain tissues (i.e. separate grey matter and white matter). Diffusion-weighted imaging (DWI) can be used to estimate microstructural properties within the white matter (Zhang et al., 2012) and to visualise the trajectory of white matter pathways (Basser, Mattiello & Le Bihan, 1994). Other sequences have been tuned to assess myelination (Glasser and Van Essen, 2011; Prasloski et al., 2012; for review see Heath et al., 2018). Using *in-vivo* recordings, the function of the brain can be assessed by measuring task-related blood oxygen level changes (BOLD; Ogawa et al., 1990; Logothetis et al., 2001) or modelling brain functional dynamics at rest (Fox and Raichle, 2007; Biswal, 2012). Compared to histology, MRI data gives access to a significantly greater number of specimens as it does not require the death (i.e. natural or sacrifice) of animals and allows to acquire complementary information on the structure and function of the brain within the same sample and can even be extended to measures of plasticity mechanisms using longitudinal designs. MRI is conveniently digital and shareable amongst researchers for easier replication of findings. MRI can also be mathematically modified (e.g. log transformation, Donahue et al., 2016) and reanalysed to address novel questions in already collected data (Balezeau et al., 2020). Finally, while acquiring and using MRI data in primates come with challenges with regards to collecting and harmonising data across species (see Milham et al., 2020 for a detailed discussion), MRI allows for the methodologically most similar cross-species comparisons (Thiebaut de Schotten and Zilles, 2019).

In this review, we summarise the emerging field of MRI-based neuroimaging of the primate brain evolution as well as gather the main scientific questions to be explored in the future.

## Brain structure

### Brain size

**Fig. 2:**
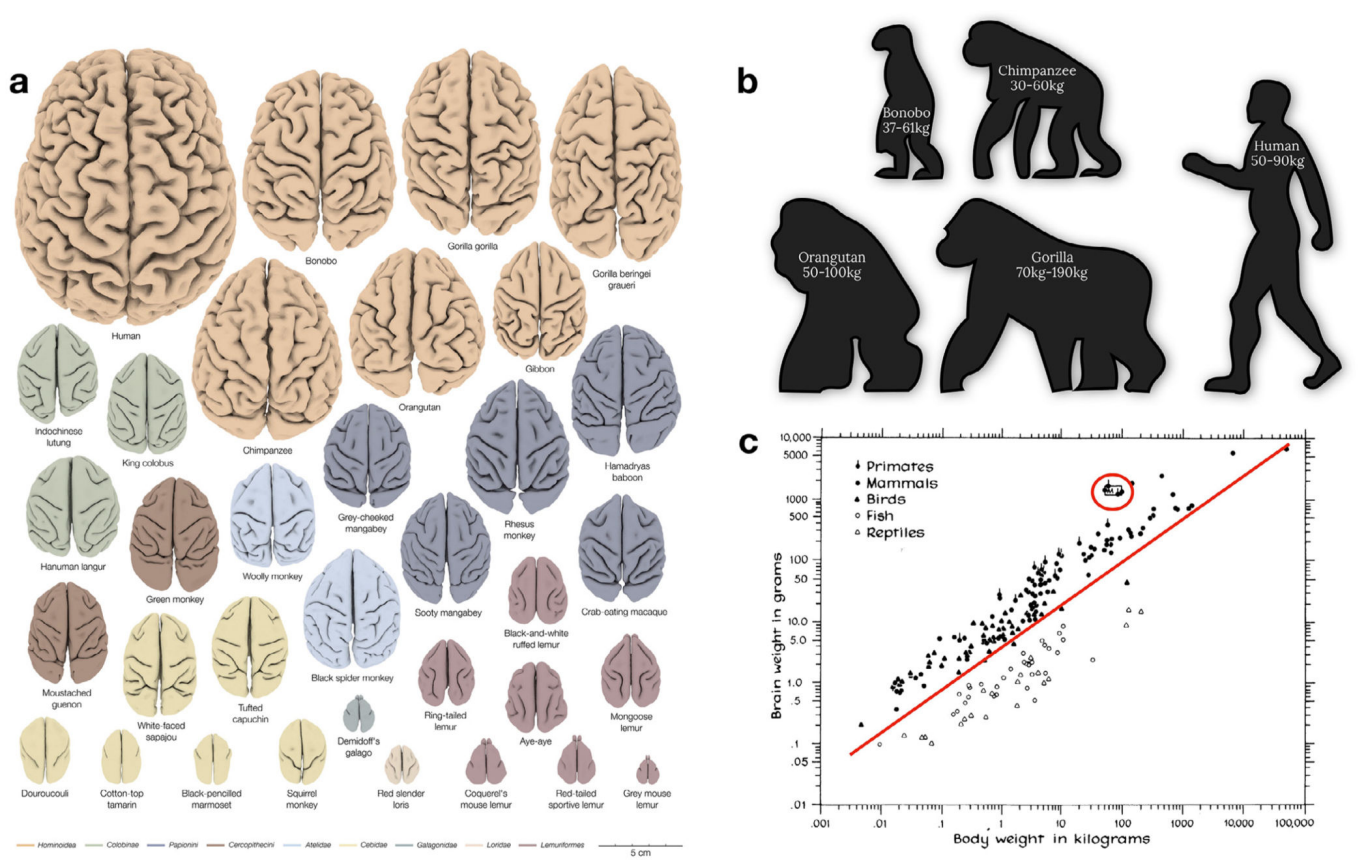
Relative brain size cross-species comparison. a) 34 three-dimensional digital brain reconstructions from the brain catalogue (Heuer et al., 2019) b) Body size and weight comparison across apes c) Brain and body weight scatter plot comparison (Jerison 1975). Note that the red circle indicates human primates who deviate from the linear relationship existing between body and brain weight.

The arguably most apparent and seemingly systematic change that occurred over primate brain evolution is the increase in total brain size relative to body size (Fig. 2). The effect of evolutionary expansion in relation to brain size is, however, not equally distributed across brain structures. This disproportional composition of the brain led to investigations of allometric rules of brain evolution. The size of one region can vary consistently with the size of another structure within the brain (Butler and Hodos, 2005). In this context, allometry refers to the study of the different pace of expansion of brain regions (Montgomery et al., 2016).

Allometric changes may provide a window for understanding the adaptations of specific neural systems in response to evolutionary pressure (Willemet, 2019; Finlay et al., 2001). In this regard, evolutionary psychology and neuroscience suggest that allometry arises from evolutionary developmental constraints, as a brain adjustment to optimise its functional organisation (Montgomery 2013; Willemet, 2015a, Montgomery et al., 2016). Along these lines, some authors have argued that similar patterns of allometric slopes across brain regions can be used to identify different grades of evolution. For instance, it has been argued that across different primate species, the prefrontal cortex shows allometric scaling with the visual cortex size. Importantly, this scaling factor is different in apes compared to monkeys (Passingham and Smaers, 2014). This finding suggests that the same developmental constraints happened across all primates, but that a major adaptive change separates monkeys from apes.

Due to the limited availability of tissue, comparative studies often rely on small samples and many studies still rely on antiquated datasets. These datasets often did not delineate different cortical territories with high accuracy, leading to fierce debates (e.g., Passingham and Smaers, 2014; Barton and Venditti, 2013). These limitations impede on our ability to assess within-species diversity accurately and might have biased our understanding of between-species differences. The ability of neuroimaging to acquire data from multiple individuals per species and imaging sequences might allow a more representative parcellation of the brain (Van Essen et al., 2011, 2016 Donahue et al., 2018). Such an approach will benefit allometric studies by providing better quantitative measurements and replicable findings as well as improve the granularity of investigations.

### Gyrification

Typically, primates with smaller brains show a smoother, less convoluted brain surface than species with larger brains (see Fig. 2a; Hofmann, 2012; Heuer et al., 2019). The level of convolution of the cortex, also called gyrification index, is easily quantifiable with surface-derived MRI measurements. A convoluted cortex allows for more surface area to be packed into the limited volume within the skull (i.e. linear scaling between surface area and brain volume; Prothero and Sundsten, 1984) providing more space for grey matter cell bodies, white matter connections, and glial cells (Namba and Huttner 2017). The convolution of the cortex (i.e. gyrification) would occur because of an imbalance in the expansion of cortical (i.e. outer layer) and subcortical layers (i.e. inner layer) of the brain (Richman, 1975; adapted by Lui et al., 2011). Computational modelling of an imbalance between inner and outer layer growth success-fully reproduced a folding pattern similar to the mammalian brain (i.e. buckling shell models, Toro and Burnod 2005; Toro 2012; Bayly et al., 2013; Tallinen et al., 2014, 2016; Foubet et al., 2019). Biologically, the cortico-subcortical imbalance would be due to the tangential migration (Lui et al., 2011; Reillo et al., 2011), and the radial intercalation of neurons during development (i.e. pushing of neighbouring neurons in the outer cortical plate aside, Striedter et al., 2015). With evolutionary expansion, a disproportional expression of these biological mechanisms could explain increased cortical folding (Mota and Herculano-Houzel 2015; Amiez et al., 2019). This latter hypothesis partially implies that the dynamic relationship between brain expansion and gyrification during early stages of brain development differ across species. However, investigating such relationships across the brain developmental stages is more likely achievable by means of comparative “longitudinal” imaging during brain development, which is typically be unthinkable with standard histological methods but is feasible with MRI (Rabiei et al., 2017). While models based on the ideas described above are successful in producing random folding patterns, they do not explain why folding patterns show similarities across the brains of the same or even different species (see Fig. 2a).

Hypothetically, similarities in folding patterns could be related to preferences for neurons to migrate in cortical areas (i.e. proliferation hotspots; Retzius, 1896; Kriegstein et al., 2006) and genetically coded. If this assumption is correct, combining genetic measurements with cortical folding patterns derived from neuroimaging in the future should offer some novel insights. Recent evidence already demonstrates a significant relationship between brain surface and genetics in humans in a collaborative cross-laboratory dataset of more than 50,000 participants (Grasby et al., 2020) as well as its distribution over the brain (Valk et al., 2020). Extending genetic brain imaging MRI to other primates will not only validate this work but also shed light on the main brain surface evolutionary mechanisms.

While the placement of proliferation hotspots may as well be determined genetically, other authors pointed out that the mechanical formation of folding leads to a complex stress influencing the stiffness of the cortex (e.g., Foubet et al., 2019). The resulting differences in stiffness might potentially influence the migration of neurons during brain development (Franze, 2013). Therefore, initial folding as proposed by the buckling shell models might be sufficient to create the intricate folding pattern as seen in mammalian cortices, which in turn, may lead to the observed pattern of regional cell composition and neural connections (Heuer and Toro, 2019). Reversely, another hypothesis suggests that the stereotypical pattern of folding would come from the tension applied by the axons on the cortex (i.e. axonal tension hypothesis; Van Essen, 1997). In this theory, tangential forces that are created by the tension along obliquely oriented axonal trajectories induce folds at specific locations. As genetic molecular gradients drive axonal migration during brain development (Krubitzer, 2007; Renier et al., 2017), the future combination of cortical folding estimate, white matter diffusion imaging tractography and genetic measurements across species may reveal a tripartite relationship between these factors.

### Sulcal anatomy

A prominent anatomical feature on the primate brain is the presence of folds, or sulci (Fig. 2a). Even though folding patterns may appear to vary greatly, even between individuals of the same species, sul-cal organisation is not at all random, and adheres strongly to a topographical organisation (Petrides, 2012). Anatomically, sulci often constitute borders between cytoarchitectonic areas (White et al., 1997). For instance, across human (Penfield and Boldrey, 1937) and non-human mammal brains (Ferrier, 1873), the central sulcus serves as the border between the motor cortex and the somatosensory cortex. Function-wise, a growing body of work has demonstrated precise relationships between an individual’s local sulcal morphology and the location of functional areas including the sensorimotor cortex (e.g. Zlatkina et al., 2016; Germann et al., 2020), prefrontal cortex (e.g. Loh et al., 2020; Lopez-Persem et al., 2019; Amiez and Petrides, 2018), cingulate cortex (Amiez and Petrides, 2014) and the temporal cortex (Bodin et al., 2018). This robust correspondences with the anatomical-functional organisation of the brain allows for the sulcal organisation to guide our interpretation of neuroimaging data. Especially since brain sulci are still often used as critical anatomical landmarks for navigating the brain during human and non-human primate brain surgeries. Also, most surface-based registration methods use sulci either explicitly (Auzias et al., 2013) or implicitly via geometrical maps such as curvature that are indicators of folding (Robinson et al., 2014).

**Fig. 3:**
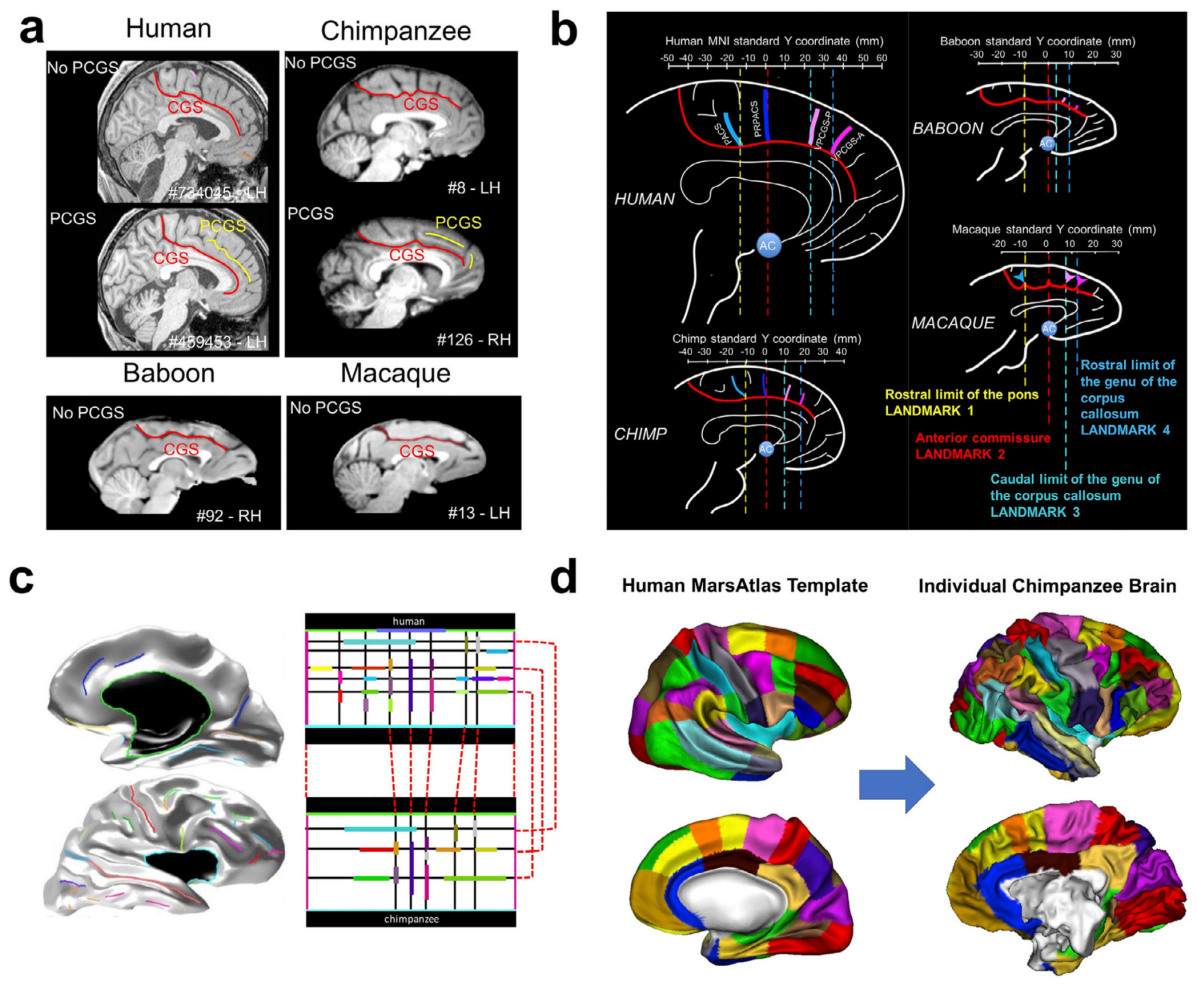
Sulcal anatomy for inter-primate brain comparisons. a) Emergence of the para-cingulate sulcus (PCGS) the primate medial frontal cortex (Amiez et al., 2019 : non-existing in baboons and macaques, but sometimes present for great apes and humans. b) Sulcal landmarks in the primate medial frontal cortex (Amiez et al., 2019). c) Projection of human brain sulci (left) onto a rectangular sulcal model (top right). Correspondences are defined between the human rectangular cortical sulci model and its chimpanzee equivalent (bottom right). d) Application of the model correspondences to map a human surface-based brain atlas onto an individual chimpanzee surface (Coulon et al., 2018).

In the primate brain, some brain sulci are conserved across species. These typically include primary sulci such as the central sulcus, superior temporal sulcus, cingulate sulcus, and the calcarine sulcus. The relationship between these primary sulci and the location of anatomical/functional regions appear to be conserved across species. For instance, the somatotopic organisation along the dorsal-ventral extent of the central sulcus, as well as along the rostro-caudal extent of the cin-gulate sulcus appears to be highly conserved across the primate lineage (Procyk et al., 2016; Loh et al., 2018). This indicates that we can potentially anchor the brains of various primate species on the basis of homologous sulcal landmarks, to perform interspecies comparisons on brain structure. In Amiez et al., 2019, this principle has been implemented to reveal the evolutionary trajectories of the medial frontal cortex across macaques, baboons, chimpanzees, and humans (Fig. 3a, 3b). This work demonstrated that, unlike previously thought the paracingulate sulcus is not a human specific feature and could be observed in chimpanzees (Amiez et al., 2019; Fig. 3a). The lateralisation of these sulci, however, is only observed in humans, which suggests further hemispheric specialisation in humans since the last common ancestors to humans and great apes (Croxson et al., 2018). As shown in Fig. 3b, by aligning brains on the basis of common sulci landmarks, the evolutionary changes in the primate medial frontal cortex become apparent and quantifiable.

Inter-species sulcal-based brain alignment can be implemented and tested using model-driven cortical surface matching (Fig. 3c, 3d). For a given species, after building a model of relative positions, orientations, and alignment of sulci in a rectangular domain, any individual cortical surface can be registered to this rectangular model, which leads to an explicit matching of different cortical surfaces based on their sulci (Auzias et al., 2013). For two different species, two different rectangular models can be built, and inter-species sulcal correspondences can then be used to define a homology between the two models (Fig. 3c). This allows for a correspondence between inter-species cortical surfaces, which can be used to compare these species and warp information from one species to the other (Coulon et al., 2018; Fig. 3d).

A key challenge to this approach is the great inter-individual variability of sulci morphology. More work will be necessary to (1) characterise the morphological variability of the various sulci in the primate brain, (2) to determine the relationship between these variations and the localisation of anatomical and functional areas, and lastly, (3) to establish the sulcal homologies between the various species. Such work would only be achievable by means of comparative MRI and data sharing in order to gather enough data to model evolutionary trends (with a minimum of three species; e.g. Balezeau et al., 2020) and quantify variability appropriately (with a minimum of 10 sample per species; Croxson et al., 2018).

### Brain connectivity

Brain areas interact in order to orchestrate cognition and behaviour. Short (local) and long (distant) connections link neighbouring and remote brain regions to facilitate these interactions. Importantly, exploring connections informs about the organisational principles of the information processing in the brain (Mars et al., 2018) and is one step closer to explaining the functioning of the brain (Takemura and Thiebaut de Schotten 2020). Therefore, studying the extent to which evolutionary changes in brain structure entail specific differences in brain connectivity is a current agenda in comparative neuroscience. Neuroimaging is ideal for this purpose, as there are many imaging techniques available to elucidate various aspects of connectivity. Hence, connectivity is one of the most developed areas of comparative neuroimaging (Goulas et al., 2014; Miranda-Dominguez et al., 2014; van den Heuvel et al. 2016).

Brain connectivity can be assessed either by reconstructing structural connections (i.e. tractography based on diffusion weighted imaging) or measuring the covariation of activity across brain regions (i.e. the synchronisation of activation derived from functional MRI).

### Gross anatomy

**Fig 4:**
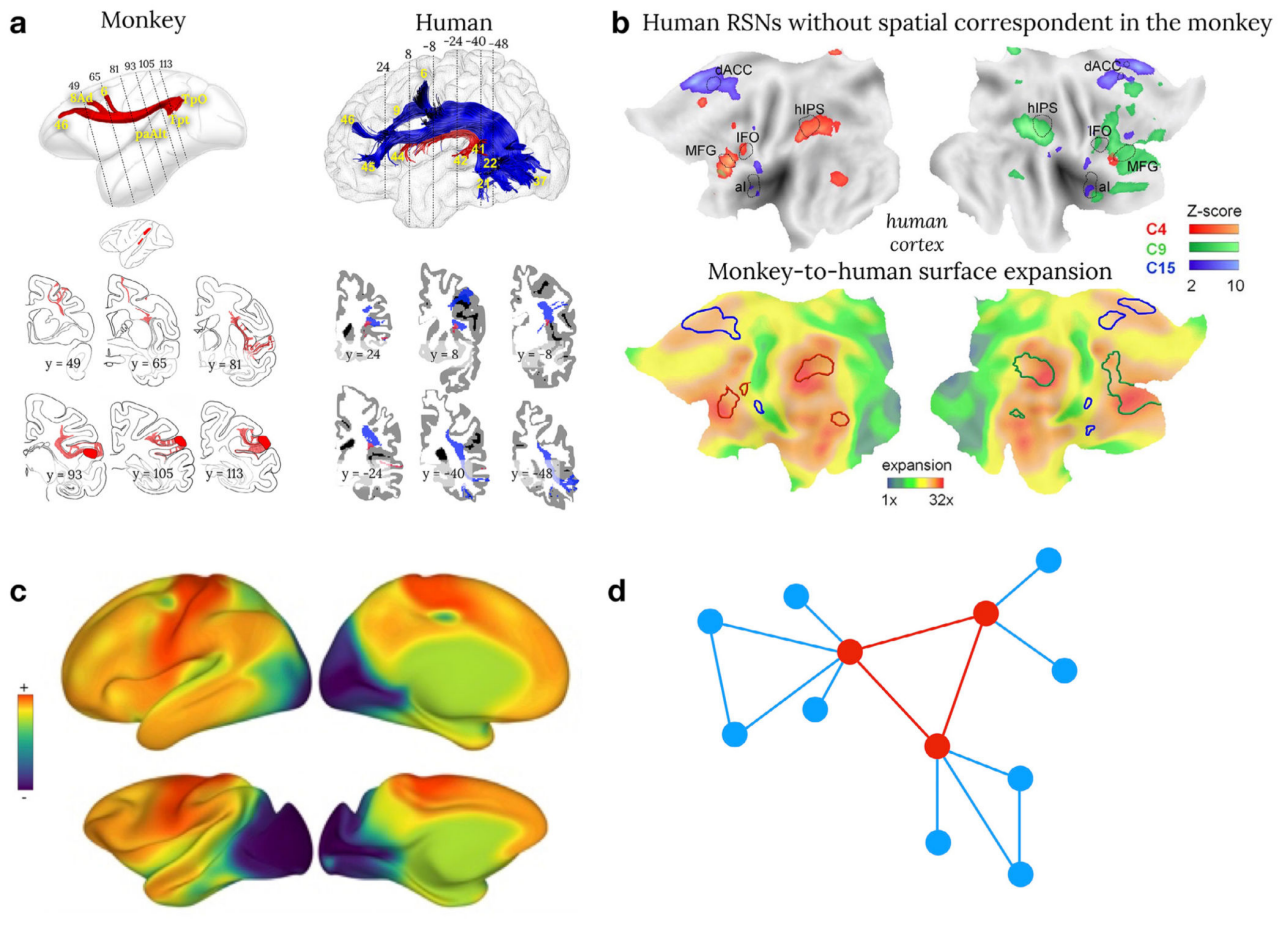
Brain connectivity cross-species comparison. a) Comparison between post-mortem axonal tracing in monkeys (cases 7&9 modified from Schmahmann and Pandya, 2006) and human in vivo spherical deconvolution tractography. Common anatomical features between human and monkey are reconstructed in red whereas anatomical differences have been coloured in blue (Thiebaut de Schotten et al., 2012) b) Flat maps of the human resting state functional connectivity without correspondence with the monkey (upper row) and its correspondence to cortical expansion maps (Mantini et al., 2013) c) Preliminary comparison of the principal gradient in humans and macaques (see Brain integration section of this paper for a definition of brain gradients ; Xu et al., 2019) d) The rich club organisation of the brain where regions in red are interconnected together and a hub for regions in blue (Bullmore and Sporns 2012).

Comparative neuroimaging has revealed gross connectivity differences between primate species (Ardesch et al., 2019; Balsters et al., 2020; Xia et al., 2019). For instance, the inferior fronto-occipital connections (Thiebaut de Schotten et al., 2012; Barrett et al., 2020) and the arcuate fasciculus (see Fig. 4a, Rilling, 2008; Thiebaut de Schotten et al., 2012; Eichert et al., 2018; Barrett et al., 2020; Balezeau et al., 2020) are more prominent in humans than in monkeys. The arcuate fasciculus has sparked interest, in particular, because of its fundamental role in human language processing (Barbeau et al., 2020; Balezeau et al., 2020). In addition, the parietal lobe has been linked to uniquely human functions and the underlying white matter has some prominent structures in humans but also some unique connections in monkeys (Catani et al., 2017). The functional interactions between the frontal and parietal lobes are also more prominent in humans than in macaques (see Fig. 4b, Patel et al., 2015; Mantini et al., 2013; Mars et al., 2011) and might reflect evolutionary trends within the attentional networks (Patel et al., 2015). However, structural connections and functional connectivity are usually examined separately. Comprehensive models of connectivity will require the combination of structural-functional methods to fully grasp the *modus operandi* of how evolutionary pressure and adaptation might have modified the wiring and the organisational principles of information processing in the brain.

### Connectivity principles

Although species-specific features exist in connectivity, the tendency of two regions to be connected respects several organisational principles across (mammalian) species (Bullmore and Sporns, 2012; Ercsey-Ravasz et al., 2013, Horvat et al., 2016, Goulas et al., 2019, Vértes et al., 2012). In particular, two regions are more likely to display interconnections if they are adjacent to each other (Human: Betzel et al., 2016; Macaque: Kaiser and Hilgetag, 2006). They will also be more connected to each other if they share a similar microstructural composition (Pandya et al., 2015; Barbas, 2015) or connections with the same regions (Song et al., 2014). Recent efforts in human neuroimaging have revealed a seemingly overarching organisation principle (Huntenburg et al., 2018), providing a window of comparison into the features that underlie the spatial arrangement of cortical areas previously reported (Abbie 1940, 1942; Sanides 1962, 1970; Brockhaus 1940; Goulas et al., 2018). While classical studies have focused mainly on cross-species similarities in this overarching organisation scheme (Margulies et al., 2016; Goulas et al., 2019) preliminary evidence suggest these differences might be an evolutionary adaptation (Fig. 4c; Xu et al., 2019).

Since the neural system is costly in energy consumption, one core principle in the neural architecture has to be the minimisation of energy costs (Bullmore and Sporns, 2012). The brain wiring, therefore, can be expected to follow rules that minimise energy costs while maintaining a set of features that are indispensable for efficient brain functioning. The “rich-club organisation” (van den Heuvel and Sporns, 2011) represents a shared feature of brain architecture that fits this description (see Fig. 4d). Within the rich-club, few nodes act as hubs between otherwise segregated nodes and thus, facilitate efficient communication across the entire network (Sporns, 2013). Additionally, hubs tend to interconnect densely with each other. However, the unique properties of hubs come at a high price (van den Heuvel and Sporns, 2011). Cortical regions that represent hubs of the rich-club tend to show high metabolic demand and are vulnerable targets for pathogenic agents (Bullmore and Sporns, 2012; Griffa and Van den Heuvel, 2018). From an evolutionary perspective, the advantages of a rich-club organisation appear to outweigh its drawbacks. Rich-club topology is a common feature amongst various species, ranging from invertebrates to primates (van den Heuvel, Bullmore and Sporns, 2016; Rubinov et al., 2016). Another example includes the comparison of the macroscale organisation of human and macaque connectivity (Goulas et al., 2014; Miranda-Dominguez 2014). However, these promising analyses should be extended to other primates in order to establish their approximate phy-logeny.

### Brain function

Brain areas increase their activity when contributing to cognitive function, and this increase is detectable with task-related functional magnetic resonance imaging. The question about comparability between cognitive abilities is debated, for advanced functions such as communication (Mertz et al., 2019) or decision making (Tremblay et al., 2017; Fouragnan et al., 2019) as well as more primary functions such as episodic memory (Croxson et al., 2011; Pause et al., 2013) or even motor cognition (Borra and Luppino 2019). For instance, the superiority of chimpanzees over college students in a working memory task (Inoue and Matsuzawa 2007) is directly related to training (Cook and Wilson 2010) and indeed highlights the issue of comparability of functions across species. A recent alternative has been to compare functional activation related to the free viewing of video during fMRI measurements across primates (Mantini et al., 2012ab; Mantini et al., 2013; Russ and Leopold 2015; Sliwa and Freiwald 2017). However, species differences likely exist in the interpretation of the video limiting the interpretability of such interspecies differences. Therefore, more general features of brain function, such as brain lateralisation and brain integration measures, have been preferred in comparative neuroimaging paradigms.

### Brain lateralisation

In order to conserve the speed of brain oscillations across species, a functional reorganisation might have occurred (Buzsaki, Logothetis & Singer, 2013) to compensate for interhemispheric delay related to brain size (Phillips et al., 2015). Accordingly, the inter-hemispheric independence theory suggests that during evolution, the increase in brain size led to increased functional lateralisation in order to avoid excessive conduction delays between the hemispheres (Ringo et al., 1994). Functional processing asymmetries have been derived from the neuroimaging-based study of the corpus callosum (Friedrich et al., 2017; Karolis et al., 2019; Horowitz et al., 2015) and hemispheric asymmetries (Hopkins 2015; Margiotoudi et al., 2019; Eichert et al., 2019; Marie, 2018; Thiebaut de Schotten, 2011; Amiez et al., 2019). Accordingly, an increase in functional lateralisation should be associated with a decrease of corpus callosum size or density as well as an increase in anatomical asymmetries. However, a comprehensive study of functional lateralisation across primate brains is still missing due to the scarcity of appropriate data. Lateralised patterns in tracing studies and cytoarchitectonic maps from macaques and marmosets, for instance, are rarely investigated for understandable ethical and financial reasons. As a consequence, both hemispheres are usually considered as equal limiting this line of research. An HCP-like multimodal neuroimaging approach would enable addressing brain lateralisations at the microarchitecture, connectomics, and functional levels as well as their interdependencies. For instance, in the orbitofrontal cortex, even when both hemispheres are similar at the cytoarchitectonic level (Mackey and Petrides, 2010), rs-fMRI analysis can reveal hemispheric differences in connectivity within the default-mode network (Lopez-Persem et al., 2020), in both humans and macaques. Studying the evolution of brain lateralisation in primate models would benefit from the reuse of MRI data progressively made available thanks to new open data initiatives (Milham et al., 2018, 2020). This will allow to compare larger numbers of species and to disentangle true species differences from individual noise.

### Brain integration

The brain processes incoming sensory information (e.g. auditory and visual) along processing streams towards increasingly abstract and integrative, or *associative*, levels (Pandya and Yeterian 1990). Before comparative neuroimaging, post-mortem studies already suggested that cortical areas related to association processes are enlarged in humans compared to other primate species (Schoenemann, 2006; Van Essen and Dierker, 2007; Hrvoj-Mihic et al., 2013; Hofman, 2014). The pre-frontal lobe has been specifically explored, comparatively, with regards to expansion (Sherwood et al., 2005; Semendeferi et al., 2002, 2001 Petrides et al., 2012; Hofman 2014), cytoarchitecture (Palomero-Gallagher et al., 2013; 2019) and relative scaling of white matter (Smaers et al., 2010; Barrett et al., 2020). Bryant et al. (2019) extended this work to the visual and auditory systems. They investigated the connections of the primary and secondary processing areas of the visual and auditory cortex. In humans, chimpanzees and macaques, the connectivity of the primary visual cortex showed a retinotopic organisation with its association area. However, the primary visual cortex had additional connections to the temporal pole only in humans and chimpanzees. Quite similarly, the primary auditory cortex showed a gradual increase of connection with temporal associative cortex in chimpanzee and humans, but not in the macaque. These results suggest that a gradual expansion of the associative cortex between the auditory and visual cortices must have occurred along the chimp-human phylogenetic lineage. In line with these gradual changes, the advent of advanced MRI analyses has enabled the comparison of a variety of other properties such as the “principal gradient” of connective properties (Margulies et al., 2016; Buckner and Margulies, 2019), which summarise a specific functional connectivity signature distributed across the human brain (Huntenburg et al., 2018).

While the idea of organising the brain in terms of gradients is relatively new in neuroimaging, the concept itself has been evinced across modalities and species for more than 100 years (Vogt and Vogt, 1919; Flechsig 1920; Hopf, 1954a,b, 1955, 1956, 1968a, 1969, 1970b). In a broad perspective, comparative neuroimaging could provide a systematic assessment of the structural variation between cortical areas in multiple species. This could test whether this organisational principle is the basis of functional specialisation and evolution of brain areas, as recently suggested in rodents (Fulcher et al., 2019; Lu et al., 2012) and humans (Waymel et al., 2020).

Overall, these results are encouraging in our endeavour to understand the differences in the structure of the brain and its functions across species. However, it is important to stress that the same network in different species can have different dynamic properties and potentially different functions (Mantini et al., 2013). Consequently, similarities in brain organisation across species should not be considered as entirely equivalent brain functions.

## Perspectives & future directions

Imaging the primate evolutionary tree would be a new stepping-stone for neuroscience. Access to more data across species will allow us to model the brains of common ancestors by extrapolating from the wealth of information on commonalities and divergences between species, families, orders, and classes. Having access to all levels of primate entities will allow us to create reference spaces, which in turn may grant better methods for inter- and intra-species comparisons. Ultimately, these developments can help us to form a true ‘neuroecology’ of different brains (Mars and Bryant, in press). In other words, we would be able to understand how a given brain is adapted to fit its environmental niche within the constraints of its evolutionary history.

The resources and methodologies outlined above not only allow for further investigation of primate evolution but can be extended to address crucial questions about similarities and differences in other mammalian species, including humans. The mouse is currently the most commonly used mammalian model in scientific research (Dietrich et al., 2014), and the species for which the most detailed mapping of a wide range of cellular and anatomical brain properties has been obtained. Yet, there is still limited consensus on how primates and rodents differ in terms of their brain structure and connectivity. Employing the MRI-based methodologies outlined above, Balsters et al. (2020) showed an 69-80% overlap in cortico-striatal connectivity fingerprints for humans and macaques compared to a meagre 15% overlap between humans and mice and a 31% overlap between mice and macaques. Given the prevalence of animal models in biomedical research, it is of paramount importance that neuroecology understand the differences between primates, both human and nonhuman, and other species such as rodents.

Whilst primates share some cognitive abilities such as visual perception and motor functions, as well as many emotional processes, their underlying neurobiology may differ. These similarities in function are typically investigated with the assumption that shared traits between primates (i.e. homology) are inherited from a common ancestor—divergent evolution. Most comparative neuroimaging studies investigate closely related species and hence examine divergent evolution. In more distant species, however, another process may have led to the onset of a similar function— “convergent evolution”. This so-called convergent evolution postulates that similar functions in distantly related species evolved independently from each other as a result of evolutionary pressure to adapt to similar environmental or ecological factors (i.e. homoplasy). As a consequence, both evolutionary principles, namely homology and homoplasy, can both lead to structural and/or functional similarities. Supporting evidence for homology and homoplasy is well documented in the field of genetics. For instance, many animal phyla share basic multifunctional regulatory genes such as Pax-6. This gene is involved in the development of light-sensitive cells and initiates eye formation in flies, but also frogs (Altmann et al., 1997; Halder et al., 1995). Despite the eyes of flies and frogs being homoplasies, the initiating gene is homologous. Hence, homology and homoplasy should be considered as complementary in our understanding of brain evolution and can be assessed using an extensive database of primate species only accessible through collaborative neuroimaging.

Another perspective would be to derive the brain of our phylogenetic ancestors (see Kaas, 2011; 2013 for discussion) by registering different species’ brains into a common space Although this might sound implausible, recent preliminary evidence already indicates that it is feasible (Heuer et al., 2020; https://katjaq.github.io/brainscapes). Using this possibility across proximal and more distantly related primate species may offer new insights into brain anatomy across taxonomic families, classes, and orders. Ideally, such endeavours will require the integration of multiple modalities of magnetic resonance imaging with several specimens for each primate species.

Finally, although there is much effort to identify the neural basis of species-defining cognitive functions, less research is devoted to the evolutionary processes through which those functions and their underlying neural adaptations have arisen. Questions about the evolutionary processes imply that an event, such as a genetic mutation or external evolutionary pressure, is responsible for the occurrence of adaptations. In this regard, many species share a common environment and live through competitive or collaborative interactions or in a predator-prey relationship. Therefore, the imaging of the evolutionary tree is accompanied by the intriguing opportunity to investigate the co-evolution of brain structures across interacting species and thus, investigate brain evolution from a novel neuro-ecological perspective.

## Data Availability

N/A
